# Large inter-individual and intra-individual variability in the effect of perceptual load

**DOI:** 10.1371/journal.pone.0175060

**Published:** 2017-04-13

**Authors:** Hadas Marciano, Yaffa Yeshurun

**Affiliations:** 1 The Institute of Information Processing and Decision Making, University of Haifa, Haifa, Israel; 2 Department of Psychology, University of Haifa, Haifa, Israel; University of Verona, ITALY

## Abstract

This study examined whether the recurrent difficulty to replicate results obtained with paradigms measuring distractor processing as a function of perceptual load is due to individual differences. We first reanalyzed, at the individual level, the data of eight previously reported experiments. These reanalyses revealed substantial inter-individual differences, with particularly low percentage of participants whose performance matched the load theory’s predictions (i.e., larger distractor interference with low than high levels of load). Moreover, frequently the results were opposite to the theory's predictions–larger interference in the high than low load condition; and often a reversed compatibility effect emerged–better performance in the incompatible than neutral condition. Subsequently, seven observers participated in five identical experimental sessions. If the observed inter-individual differences are due to some stable trait or perceptual capacity, similar results should have emerged in all sessions of a given participant. However, all seven participants showed large between-sessions variations with similar patterns to those found between participants. These findings question the theoretical foundation implemented with these paradigms, as none of the theories suggested thus far can account for such inter- and intra-individual differences. Thus, these paradigms should be used with caution until further research will provide better understanding of what they actually measure.

## Introduction

The perceptual load theory claims that perceptual load is one of the major factors determining the selectivity of attention [1, 2]. According to the theory, the perceptual load elicited by the relevant elements in a given task influences the ability to ignore task irrelevant elements. When the load is high, attentional resources are fully consumed by the relevant stimuli and no resources are left to perceive irrelevant stimuli. In contrast, under low levels of perceptual load not all the attentional resources are consumed, and residual attentional capacity spills over to process irrelevant stimuli. Hence, when the irrelevant stimuli–the distractors–are incompatible with the correct response they should induce a sizeable distractor interference with low levels of load, but with high load levels no interference is expected.

Marciano and Yeshurun [3] showed that high perceptual load did not always prevent distractor interference (for a full description of the relevant results see Appendix A in S1 File). Specifically, in four out of the five experiments conducted in that study larger distractor interference was found with high levels of load than with low load levels (Experiments 1, 2a, 2b, accuracy measurement, and Experiment 3, RT and accuracy measurements). Only with Experiment 4, in which the number of possible distractor locations was reduced from 10 to 2, the pattern of results expected by the load theory was replicated. Based on these findings we concluded that high levels of perceptual load increase attentional selectivity only when there is minimal uncertainty regarding the spatial location of the distractor [3]. However, this uncertainty effect on distractor interference was not replicated in a following study [4]. In three out of the four experiments (Experiments 2–4, inverse efficiency scores, for a full description of the relevant results see Appendix B in S1 File) of that later study we once again found larger distractor interference under high than low levels of load, even though there were only two possible distractor locations. Thus, this large distractor interference with high levels of load could not be attributed to high spatial uncertainty. Taken together, these findings suggest that perceptual load is not the main factor affecting the efficiency of the attentional selectivity, at least not with the paradigm employed in these experiments. Other studies also found significant distractor interference under high perceptual load (e.g., [5–9]). These studies further support the conclusion that attentional selectivity might be affected by other factors than perceptual load.

One plausible reason for such non-robust effects of perceptual load on attentional selectivity could be individual differences. If inter-individual variance is relatively high, different effects of load may emerge in different experiments depending on the specific group of individuals who participated in each experiment. The current study tested this possibility. In the first part of this study we reexamined our former data taken from the five experiments of Marciano and Yeshurun [3] and three experiments (Experiment 2–4) of Yeshurun and Marciano [4]. One experiment from the latter study (Experiment 1) was not included because its load manipulation was different from the other experiments included in the analyses. In the current paper, the data of these experiments were reanalyzed at the individual level, rather than at the aggregate level as was done in the former studies. These analyses explored the degree of individual variability in the effects of perceptual load on the magnitude of distractor interference. If perceptual load is a predominant factor determining the efficiency of attentional selectivity then the manner by which distractor interference varies as a function of load level should be similar for most of the participants. However, if a large degree of individual differences is observed this would indicate that perceptual load is not a cardinal factor. The second part of this study focused on within-participant variability. Specifically, this part examined whether the observed pattern of results is stable for a given participant across different experimental sessions. We conducted an additional experiment that was identical to Experiment 4 from Marciano and Yeshurun [3], and then seven observers participated in four more identical experimental sessions on different days. If the manner by which perceptual load affects the magnitude of distractor interference depends on some structural factor or any stable personal trait (e.g., working memory capacity), a consistent pattern of results should be found for a given participant across all sessions.

## Part I: Analysis of individual differences

### Experiments 1, 2a, 2b, 3, and 4 from Marciano and Yeshurun [3]

#### Method

***Participants***

A total of 103 participants (74 females, average age—23.7 years old, ranged from 18 to 33) took part in these five experiments (Experiment 1: 19 participants; Experiment 2a: 24 participants; Experiments 2b, 3, and 4: 20 participants, each). All the participants were students from the University of Haifa with normal or corrected to normal vision, and all were naive to the purpose of the study. In addition, none of them participated in more than one experiment.

***Stimuli***

**Fig 1:**
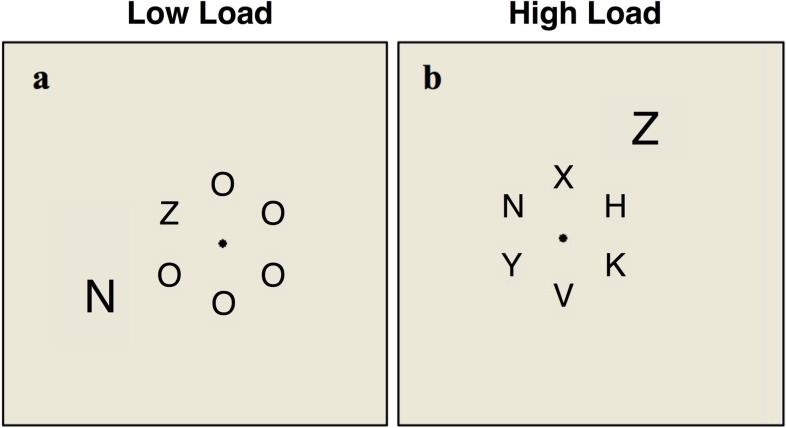
Examples of the stimuli employed in all the experiments: (a) Low load condition; (b) High load condition.

The display consisted of 6 black letters (0.6° x 0.4° each), presented on an imaginary circle (radius of 2°) against a light gray background, with a 1.7° center to center distance between two neighboring letters. One of these letters was the target. On half of the trials the target was the letter N, and on the rest of the trials it was the letter Z (Experiment 1, 2a, 3, and 4) or X (Experiment 2b). The target appeared equally often at each of the 6 possible locations. The other 5 letters were either all O's in the low load condition or X, K, H, Y, and V in the high load condition (in Experiment 2b these letters were: D, K, H, Y, and V). Additionally, the display included one distractor letter (0.9° x 0.5°, Fig 1). The distractor appeared at 4° of eccentricity, in one of ten possible locations around the central circle (Experiments 1–3) or one of two possible locations to the right or left of the central circle (Experiment 4). The size difference between the 6 letters and the distractor was designed to account for the effect of eccentricity [10]. In Experiment 1 the distractor was either compatible with the target (i.e., N when the target is N or Z when the target is Z), or incompatible (i.e., N when the target is Z or Z when the target is N), with equal probability. In Experiments 2a and 2b the distractor was either incompatible or neutral (i.e., T or L), with equal probability. In Experiments 3 and 4 the distractor was either compatible, incompatible or neutral, with equal probability.

***Procedure***

Viewing distance was held fixed at 57 cm with a chin-rest. The task was to indicate as quickly and accurately as possible whether the target was an X or an N in Experiment 2b or Z or an N in the rest of the experiments, while ignoring the distractor. Each trial started with a fixation point presented at the center of the screen for 1000 ms. In order to prevent eye movements (e.g., [11]), the letter stimuli followed for a short duration of 150 ms, and were replaced with the fixation point until the participant responded but no longer than 3000 ms. After responding, a 500 ms feedback was given: a '+' sign for a correct response, and a '–' sign for an incorrect response.

Experiments 1, 2a, and 2b involved four more load conditions in which the load at the periphery was also manipulated (see [3]). However, to be able to compare these experiments with those of the other study, the current analyses only included the conditions with a single peripheral letter–the distractor. Thus, with these experiments each load condition included 144 trials. Experiments 3 and 4 included three compatibility conditions presented in random order. However, the current analyses of these experiments only included the incompatible and neutral trials, as these are the critical trials in terms of the theory predictions. Thus, with these experiments each load condition included 384 trials.

#### Results and discussion

The outcomes of the repeated measure ANOVA performed on the aggregate accuracy and RT data are presented in details in Marciano and Yeshurun [3] and a full description of the relevant results is presented in Appendix A in S1 File. In sum, in each of Experiments 1, 2a, 2b and 3 a larger distractor interference emerged under high level of perceptual load with the accuracy measurement. In Experiment 3 a larger interference under high load levels also emerged with the RT measurement. Only in Experiment 4 distractor interference was found only under low level of perceptual load with both measurements, as predicted by the load theory.

**Fig 2:**
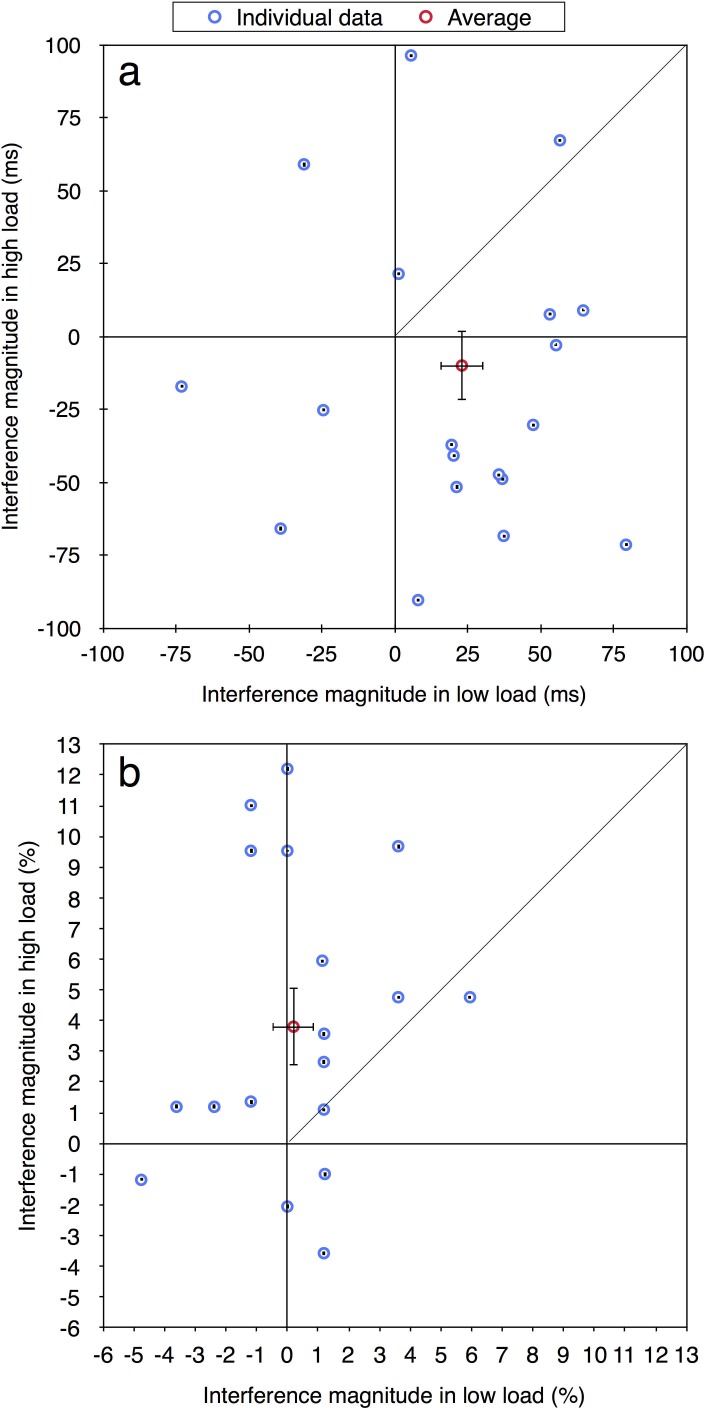
Distractor interference in the high vs. low load condition for the aggregated data (averaged across participants; in red) and for each participant (in blue) in Experiment 1: (a) RT; (b) accuracy. Error bars represent 95% confidence intervals (bootstrapped for the individual data points). Note that due to the large axes scale, individual confidence intervals are smaller than the symbol of their corresponding data point.

**Fig 3:**
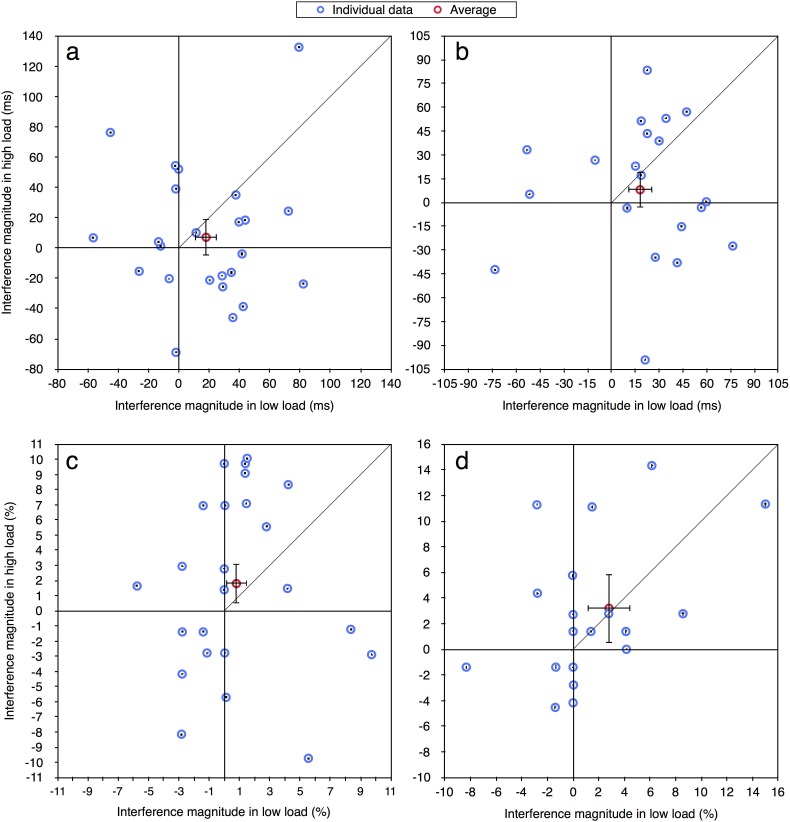
Distractor interference in the high vs. low load condition for the aggregated data (averaged across participants; in red) and for each participant (in blue): (a) Experiment 2a—RT; (b) Experiment 2b—RT; (c) Experiment 2a—accuracy; (d) Experiment 2b—accuracy. Error bars represent 95% confidence intervals (bootstrapped for the individual data points). Note that due to the large axes scale, individual confidence intervals are smaller than the symbol of their corresponding data point.

**Fig 4:**
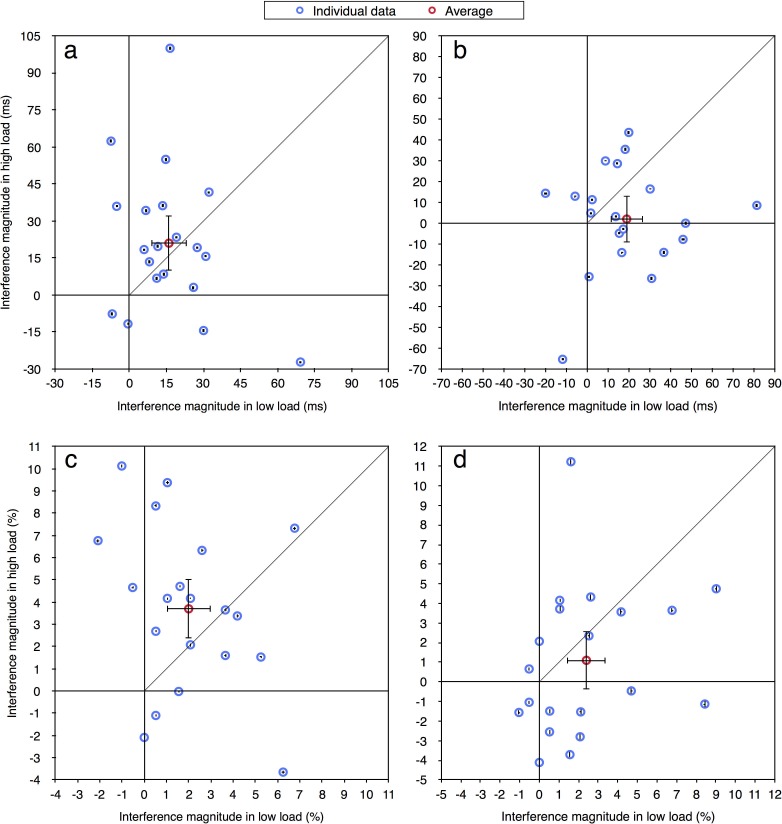
Distractor interference in the high vs. low load condition for the aggregated data (averaged across participants; in red) and for each participant (in blue): (a) Experiment 3—RT; (b) Experiment 4—RT; (c) Experiment 3—accuracy; (d) Experiment 4—accuracy. Error bars represent 95% confidence intervals (bootstrapped for the individual data points). Note that due to the large axes scale, individual confidence intervals are smaller than the symbol of their corresponding data point.

In the current paper we were interested in performance at the individual level and therefore the same data were reanalyzed. For each participant in each load condition, for both correct RT and accuracy measurements (excluding RTs shorter than 100 ms or longer than 2000 ms), we calculated the magnitude of distractor interference (Experiment 1: RT—incompatible minus compatible; accuracy—compatible minus incompatible. Experiments 2a-4: RT—incompatible minus neutral; accuracy—neutral minus incompatible). Given prior studies of distractor interference (e.g., [12, 13]) the values of these effects should be positive as an incompatible distractor is expected to impair performance. Figs 2–4 present the magnitude of distractor interference in the high load condition against the magnitude of interference in the low load condition, for all the participants of each of the experiments (i.e., each blue data point presents a single participant). The aggregated performance, averaged across all participants, is presented in red. According to the perceptual load theory, the magnitude of distractor interference should be larger with low than high levels of load; hence, it predicts that all data points should fall below the diagonal of equal magnitude. As can be seen in these figures, the individual data of all five experiments do not follow these predictions. Although some of the participants clearly demonstrated the behavior expected by the load theory, other participants showed the opposite pattern of results: smaller interference in low than high load condition. Additionally, some of the data points fall near the diagonal line (y = x), indicating that with these participants there was no difference in the magnitude of the interference between the two load conditions. Moreover, many of the data points have negative values, showing a reversed compatibility effect (i.e., better performance on the incompatible than neutral/compatible condition). Table 1 presents for each experiment the ratio and percentage of data points that fall in the first quadrant (x>0 and y>0) and below the diagonal line (y<x), thus fit the predictions of the load theory. As can be seen, in most of the experiments the percentage of participants who exhibited results that fit the load theory was higher with RTs than with accuracy, excluding Experiment 2b where this percentage was lower. Importantly, this percentage never exceeded 25%. Across all five experiments, only 15 out of 103 participants (15%) fitted the predications of the load theory for the RT measurement, and even less for the accuracy measurement (13/103, 13%). Critically, this percentage was not higher in Experiment 4 than in all the other experiments, even though this was the only experiment in that study in which the averages followed the predictions of the perceptual load theory. Indeed, when considering Fig 4B and 4D, it is evident that a similar high variability of individual data was found in Experiment 4 as in the other experiments. This discrepancy between the picture portrayed by the averages and that portrayed by individual data further underscores the importance of examining the individual pattern of results in addition to averages.

**Table 1:** Ratio and percentage of data points, in each experiment, that follow the load theory predictions (see text for details).

	Experiment 1		Experiment 2a		Experiment 2b		Experiment 3		Experiment 4	
	Ratio	%	Ratio	%	Ratio	%	Ratio	%	Ratio	%
**RT**	2/19	10	3/24	12	1/20	5	5/20	25	4/20	20
**Accuracy**	1/19	5	1/24	4	4/20	20	4/20	20	3/20	15

### Experiments 2, 3 and 4 from Yeshurun and Marciano [4]

#### Method

***Participants***

A total of 60 participants (45 females, average age—23.5 years old, ranged from 18 to 32) took part in these three experiments (Experiment 2: 24 participants; Experiments 3 and 4: 18 participants, each). All the participants were students from the University of Haifa, with normal or corrected to normal vision, and all were naive to the purpose of the study. In addition, none of them participated in more than one experiment.

***Stimuli and procedure***

The stimuli and procedure of these experiments were identical to Experiment 4 in Marciano and Yeshurun [3] (see above) except for the following. Stimuli duration was either 150 ms (as in [3]) or 100 ms. In Experiments 2 and 3 these durations were mixed within a block while in Experiment 4 the duration was fixed within a block but varied between blocks. In addition, in Experiments 2 and 4, a 200 ms mask followed the letters display. The mask was consisted of seven # symbols, each presented at one of the letters' locations.

#### Results and discussion

The outcomes of the repeated measure ANOVA performed on the aggregate data (inverse efficiency scores) are presented in details in Yeshurun and Marciano [4] and a full description of the relevant results) RT and accuracy measurements (is presented in Appendix B in S1 File. In sum, it showed that in each of these experiments a larger distractor interference was found under high than low levels of perceptual load. Thus, here, the aggregate data of all these experiments did not follow the predictions of the load theory.

**Fig 5:**
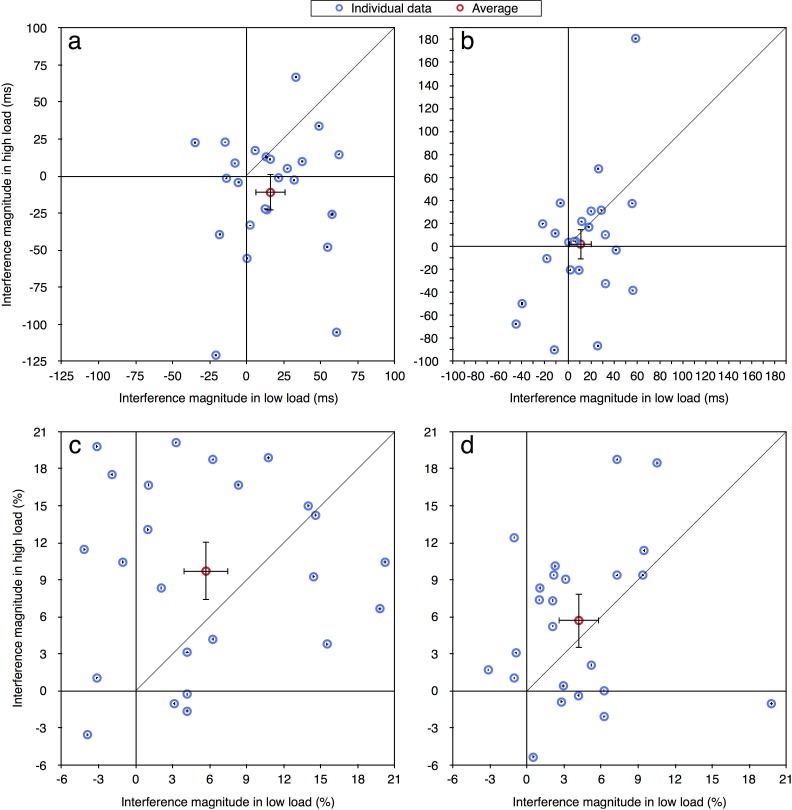
Distractor interference in the high vs. low load condition for the aggregated data (averaged across participants; in red) and for each participant (in blue) in Experiment 2: (a) 100 ms condition—RT; (b) 150 ms condition—RT; (c) 100 ms condition—accuracy; (d) 150 ms condition—accuracy. Error bars represent 95% confidence intervals (bootstrapped for the individual data points). Note that due to the large axes scale, individual confidence intervals are smaller than the symbol of their corresponding data point.

**Fig 6:**
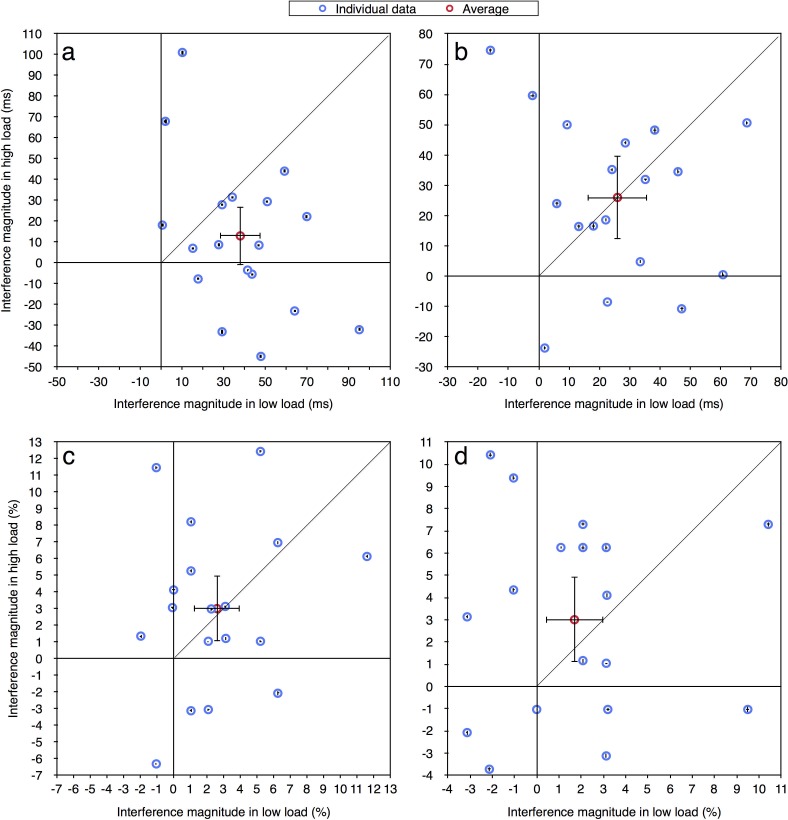
Distractor interference in the high vs. low load condition for the aggregated data (averaged across participants; in red) and for each participant (in blue) in Experiment 3: (a) 100 ms condition—RT; (b) 150 ms condition—RT; (c) 100 ms condition—accuracy; (d) 150 ms condition—accuracy. Error bars represent 95% confidence intervals (bootstrapped for the individual data points). Note that due to the large axes scale, individual confidence intervals are smaller than the symbol of their corresponding data point.

**Fig 7:**
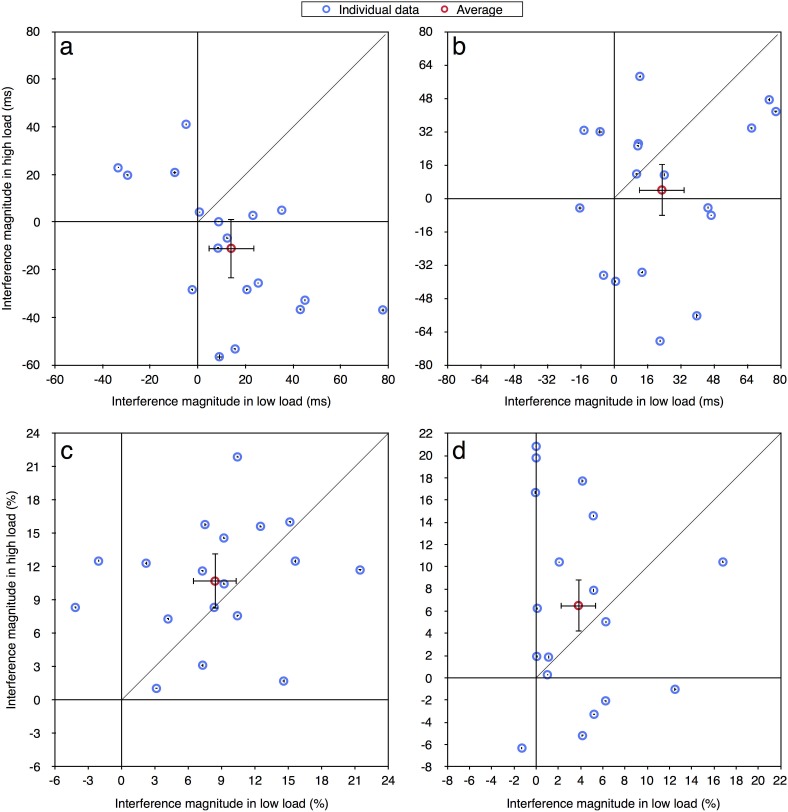
Distractor interference in the high vs. low load condition for the aggregated data (averaged across participants; in red) and for each participant (in blue) in Experiment 4: (a) 100 ms condition—RT; (b) 150 ms condition—RT; (c) 100 ms condition—accuracy; (d) 150 ms condition—accuracy. Error bars represent 95% confidence intervals (bootstrapped for the individual data points). Note that due to the large axes scale, individual confidence intervals are smaller than the symbol of their corresponding data point.

To examine performance at the individual level, we reanalyzed this data in a similar way to the analyses performed on the experiments of Marciano and Yeshurun [3]. That is, we calculated the magnitude of distractor interference for both RT and accuracy measurements (RT: incompatible minus neutral; Accuracy: neutral minus incompatible) for each participant in each load and duration condition. Figs 5–7 present distractor interference in the high load condition against that in the low load condition, for each participant in each experiment (in blue), and averaged across all participants (in red). Like before, the load theory predicts that all data points should fall below the diagonal of equal magnitude reflecting the expected larger distractor interference with low than high levels of load. However, as is evident in all figures, the data of all three experiments, regardless of durations, did not follow the theory's predictions. As with the data of Marciano and Yeshurun [3], some participants did demonstrate the behavior expected by the load theory, but other participants showed the opposite pattern of results–smaller interference in low than high load condition, and some data points fall near the diagonal line, indicating similar interference magnitude with both levels of load. Also similar to Marciano and Yeshurun [3], many of the data points have negative values, reflecting a reversed compatibility effect. Table 2 presents the ratio and percentage of data points that fit the predictions of the load theory (i.e., fall in the first quadrant below the diagonal line), for each duration condition of each experiment. Beyond all three experiments only about a quarter of the participants fitted the predications of the load theory with the RT measurement (150 ms: 15/60, 25%; 100 ms: 16/60, 27%) and the accuracy measurement when stimuli duration was 100 ms (16/60, 27%). This ratio was even smaller (8/60, 13%) with the accuracy measurement when stimuli duration was 150 ms.

**Table 2:** Ratio and percentage of data points in each experiment that follow the load theory predictions (see text for details).

	Experiment 2				Experiment 3				Experiment 4			
	150 ms		100 ms		150 ms		100 ms		150 ms		100 ms	
	Ratio	%	Ratio	%	Ratio	%	Ratio	%	Ratio	%	Ratio	%
**RT**	4/24	17	5/24	21	7/18	39	8/18	44	4/18	22	3/18	17
**Accuracy**	3/24	12	6/24	25	3/18	17	4/18	22	2/18	11	6/18	33

## Part II: Within-participant variability

Substantial individual differences under varying levels of perceptual load were reported before (e.g., [14]). Often such individual differences were attributed to a specific personality trait, skill, or cognitive capacity. For example, Sadeh and Bredemeier [15] attribute differences in the magnitude of distractor interference to differences in anxiety levels. They report that under high levels of perceptual load, participants with high levels of anxiety had larger distractor interference than those with low levels of anxiety. Similarly, Moriya and Tanno [16] report larger distractor interference under high levels of load with participants who have high social anxiety than those demonstrating low social anxiety. Bayliss and Kritikos [17] attribute individual differences in distractor interference to differences in one’s score on the Autism Spectrum Quotient (AQ). They found that individuals with more self-rated autistic traits (i.e., higher AQ scores) show a larger degree of distractor interference with high levels of perceptual load than individual with low AQ scores. Green and Bavelier [18] showed that action video-game players, who presumably have higher 'visual search' skills, demonstrated larger distractor interference with high levels of load than individuals who are not frequent video-games players. Finally, working memory capacity was also suggested to be a critical factor mediating individual differences in the selectivity of attention (e.g., [19]). Specifically with regard to perceptual load, Shipstead, Harrison, and Engle [20] found that individuals with low working memory capacity show larger distractor interference under low levels of perceptual load than those with high memory capacity.

The above studies suggest that the individual differences we and others have found might be due to differences in some relatively fixed personality trait or well established skills. If so, the pattern of results should be quite stable for a given participant. To test this possibility, we conducted another experiment that was highly similar to our previous experiments. Twenty participants took part in an initial experimental session, and then seven participants out of the 20 participated in additional four identical experimental sessions. Three of these seven participants, were chosen based on the fact that each one of them exhibited in the first session a different pattern of load effects on distractor interference. The other four participants were randomly assigned with no selection criteria. If individual differences observed in the first session are indeed stable, as expected if they are due to a personality trait or fixed cognitive capacity, then we should find for each one of these seven participants a consistent pattern of results in all five sessions.

### Current experiment

#### Method

***Participants***

Twenty students from the University of Haifa participated in the first session of the experiment (19 females; averaged age 24.8, ranged from 19 to 33). All had normal or corrected to normal vision and all were naive to the purpose of the study. None of them participated in former similar experiments. Out of these 20 participants seven participants were chosen to take part in four more identical experimental sessions. Altogether each one of these seven participants took part in five identical experimental sessions. Four of these seven participants were assigned randomly, and the additional three participants were chosen because they exhibited different patterns of results in the first session. More details regarding these three participants are given below in the results section. This experiment (as well as those reported previously) have been approved by the Ethics Committee of the University of Haifa, and was conducted according to the principles expressed in the Declaration of Helsinki. All participants singed a consent form before the beginning of the experiment.

***Stimuli and procedure***

The stimuli and procedure were identical to those of Experiment 4 in Marciano and Yeshurun [3] mentioned above.

#### Results and discussion

***Averaged results of the first session***

First we analyzed the aggregate performance of all 20 participants, which performed the first session, to test whether the results of Experiment 4 in Marciano and Yeshurun [3] will be replicated here.

***RT analysis***

**Fig 8:**
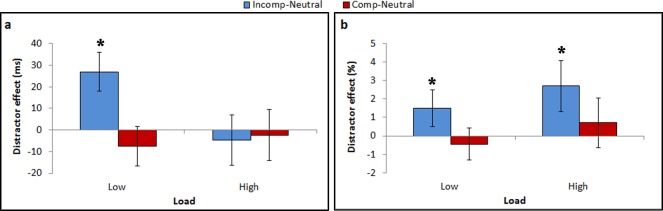
Distractor interference (incompatible vs. neutral) and distractor facilitation (compatible vs. neutral) as a function of load in the first session of the current experiment. (a) RT; (b) accuracy. '*' indicates significant distractor effect.

A two-way repeated measures ANOVA, load (low vs. high) x compatibility (incompatible, compatible, and neutral), was conducted on mean correct RT data. Trials with RTs shorter than 100 ms or longer than 2000 ms were excluded from the analysis (0.9% from the total number of trials). The main effect of load was significant, [F(1, 19) = 41.78, p<0.0001; η_p_^2^ = 0.69]; RTs were longer with high than low load (754 ms vs. 620 ms, respectively). The main effect of distractor compatibility was also significant [F(2, 38) = 9.36, p<0.0006; η_p_^2^ = 0.33]; LSD post hoc analysis indicated that RTs of the incompatible condition (696 ms) were significantly longer than the two other compatibility conditions (compatible: 680 ms, p<0.0001; neutral: 685 ms, p<0.002). The interaction between load and compatibility was also significant, [F(2, 38) = 20.23, p<0.0001; η_p_^2^ = 0.52] (Fig 8A and Table 3). LSD post hoc analysis indicated that the predictions of the perceptual load theory were met: The difference between the incompatible and neutral conditions was significant in the low load condition (p<0.0001), but not in the high load condition (p = 0.30). The difference between the distractor interference (incompatible vs. neutral) in the low vs. high levels of load was significant (t(19) = 4.52, p<0.0003, Cohen's d = 1.43). The difference between the compatible and neutral conditions was not significant in both load conditions (low: p = 0.09; high: p = 0.59).

**Table 3:** Mean correct RT, accuracy, and distractor interference (incompatible vs. neutral) as a function of load and distractor compatibility in the first session of the current experiment.

		Distractor Compatibility			Distractor interference
		Neutral	Compatible	Incompatible	
**Low load**	**RT (ms)**	614	606	641	27
	**Accuracy (%)**	95.6	96.0	94.1	1.5
**High load**	**RT (ms)**	757	754	752	-5
	**Accuracy (%)**	90.3	89.6	87.6	2.7

***Accuracy analysis***

A similar analysis was conducted on mean accuracy data. Trials with RTs shorter than 100 ms or longer than 2000 ms were excluded from the analysis (0.9% from the total number of trials). The main effect of load was significant [F(1, 19) = 45.47, p<0.0001; η_p_^2^ = 0.71]; accuracy was lower with high than low load condition (89.14% vs. 95.23%, respectively). The main effect of distractor compatibility was also significant, [F(2, 38) = 14.12, p<0.0001; η_p_^2^ = 0.43]. LSD post hoc analysis indicated that the accuracy in the incompatible condition (90.83%) was significantly lower than in the other compatibility conditions (compatible: 92.80%, p<0.0005; neutral: 92.93%, p<0.0002). The interaction between load and compatibility was not statistically significant (F<1, Fig 8B and Table 3). Nevertheless, we analyzed planned comparisons because the load theory has clear predictions concerning the simple pairwise comparisons. These planned comparisons revealed effects that are inconsistent with the theory. In contrast to the load theory’s predictions, the distractor interference (incompatible vs. neutral) was significant in both load conditions (low load: p<0.05, high load: p<0.0007), and in fact it was smaller in the low load than high load condition, though this difference did not reach statistical significance (p = 0.2371). There was no significant difference between the compatible and neutral conditions regardless of the level of load.

Thus, contrary to the pattern of results found in Experiment 4 of Marciano and Yeshurun [3], which fitted the load theory's predictions with both RT and accuracy measurements, in the current experiment the RT results followed the theory, but the results with the accuracy measurement were not consistent with the theory's predictions. Given that this experiment was identical to Experiment 4 of Marciano and Yeshurun's [3], the different findings of these two experiments are likely due to individual differences.

**Fig 9:**
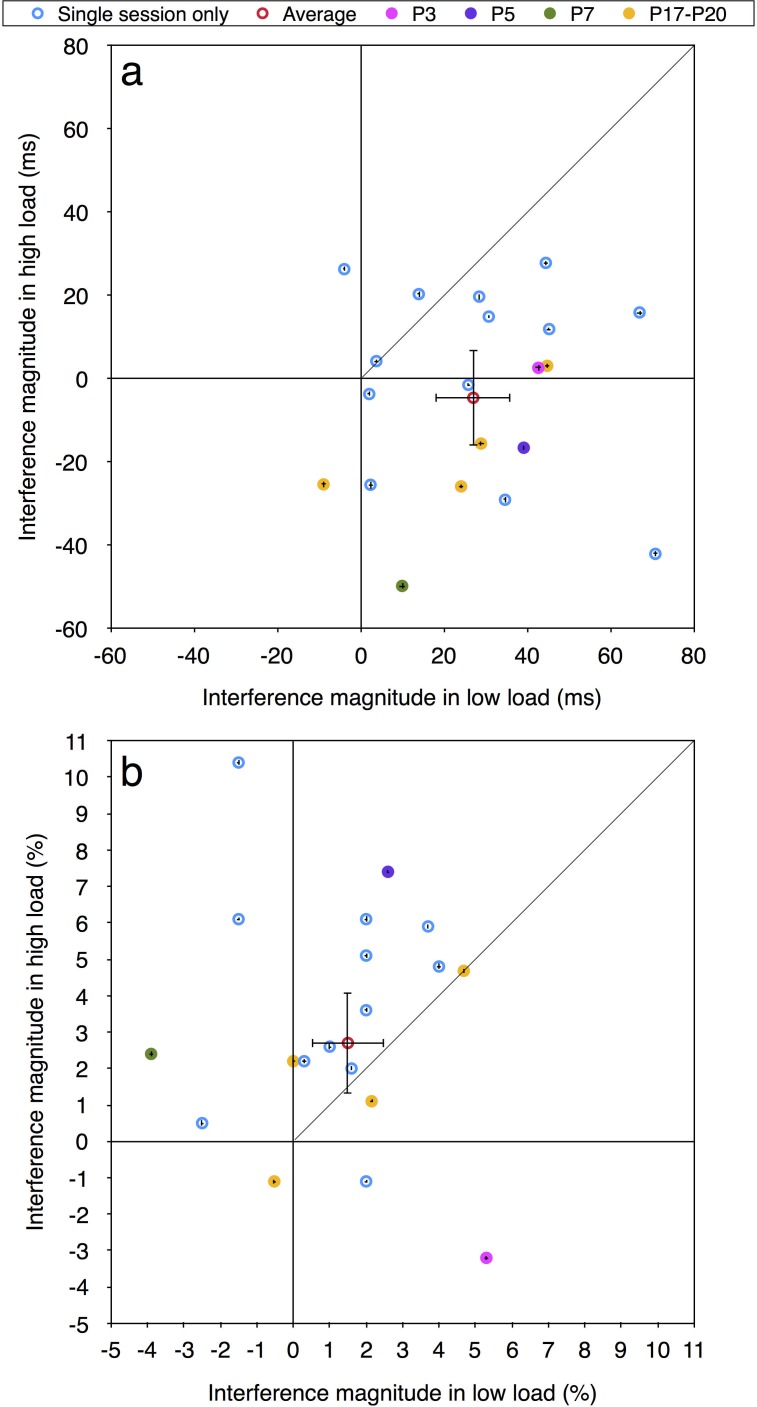
Distractor interference in the high vs. low load condition in the first experimental session of the current experiment: (a) RT; (b) accuracy. Aggregated data is in red. Data points for the 13 participants who only participated in this first session are in blue. The seven participants who participated in additional experimental sessions are marked with other colors (see text for details). Error bars represent 95% confidence intervals (bootstrapped). Note that due to the large axes scale, individual confidence intervals are smaller than the symbol of their corresponding data point.

***Individual performance—first session (20 participants)***:

Fig 9 presents the individual performance of all 20 participants who took part in the first session of the current experiment. As can be seen in this figure, large individual differences also emerged in the current experiment. With regard to the accuracy measurement, only one of the participants followed the predictions of the load theory. The majority of the data points (10 out of 20, 50%) are located in the first quadrant above the diagonal line, exhibiting pattern of results which are opposite to the load theory's predictions. With the RT measurement, seven of the participants did follow the theory's predictions (35%), one participant showed an opposite pattern of results to that predicted by the theory, and the other participants exhibited different patterns of results. Comparing these data to the individual data of Experiment 4 of Marciano and Yeshurun [3] (see Fig 4B and 4D, and Table 1) it is evident that in Experiment 4 more participants followed the theory's prediction with the accuracy measurement but fewer followed it with the RT measurement.

***Individual results in each session of the seven chosen participants***:

As mentioned above, seven participants took part in four additional sessions. Three of these seven participants were chosen because they exhibited, in the first session of the current experiment, three different patterns of results. The aim was to test whether these different performance patterns are stable. One participant (Participant 3 –P3; marked in magenta in Fig 9) showed larger distractor interference with RT under low than high perceptual load, as predicted by the load theory. Another participant (Participant 5 –P5; marked in violet in Fig 9) showed larger distractor interference with accuracy under high than low perceptual load, opposite to the predictions of the load theory. The third participant (Participant 7 –P7; marked in green in Fig 9) did not fit any specific pattern, demonstrating reversed compatibility effect (worse performance with neutral than incompatible condition) under high load with RT and under low load with accuracy. The selection process of these participants led to an uneven time intervals between sessions. The time interval between the first and the second experimental sessions was quite large (37, 62, and 29 days for participants 3, 5, and 7, respectively). For sessions 2–5, the majority of the intervals between two subsequent sessions ranged from one to eight days, although two of the participants also had another larger gap between two subsequent sessions (27 days between sessions 4 and 5 of Participant 3, and 36 days between sessions 3 and 4 of Participant 5). To ensure that these variations are not the reason for the large intra-individual differences reported below, four additional participants were chosen randomly to participate in 5 experimental sessions with more careful control over the time intervals between sessions (Participants 17–20 – P17-P20, marked in orange in Fig 9). Specifically, all 5 sessions of these 4 participants occurred within about 10 days. The maximum time interval between two subsequent sessions was four days. In addition, the time of the day at which the session was held was relatively constant for a given participant.

**Fig 10:**
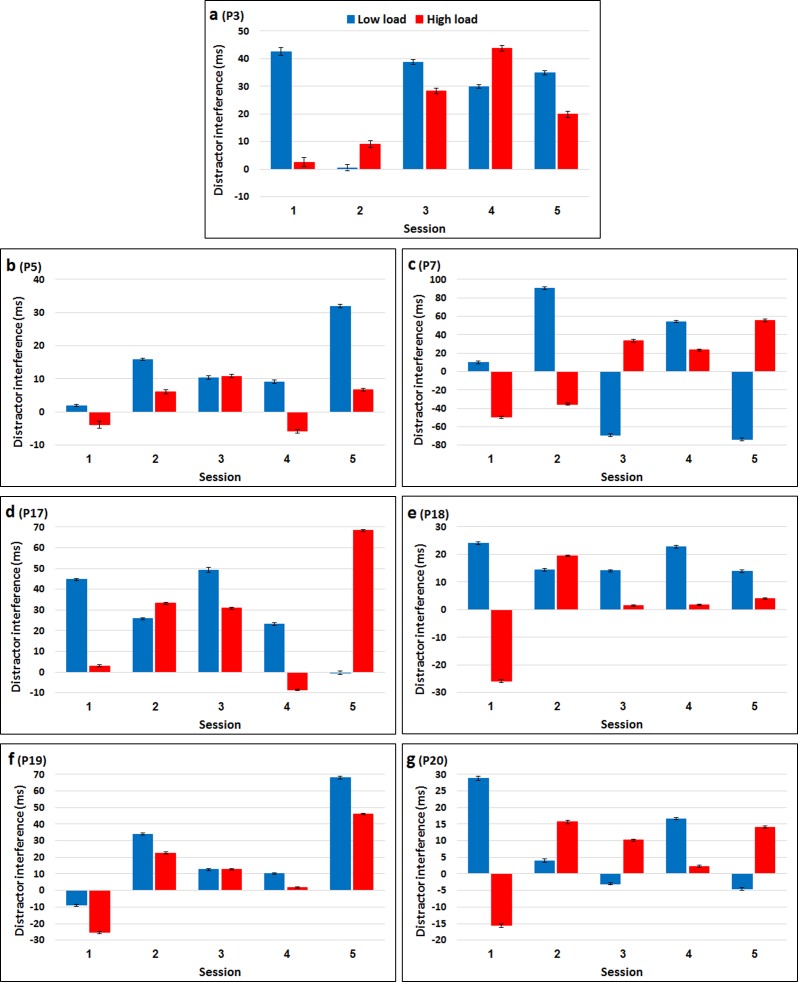
RT distractor interference (incompatible vs. neutral) as a function of load condition and session for each of the seven participants. Error bars represent 95% confidence intervals (bootstrapped).

**Fig 11:**
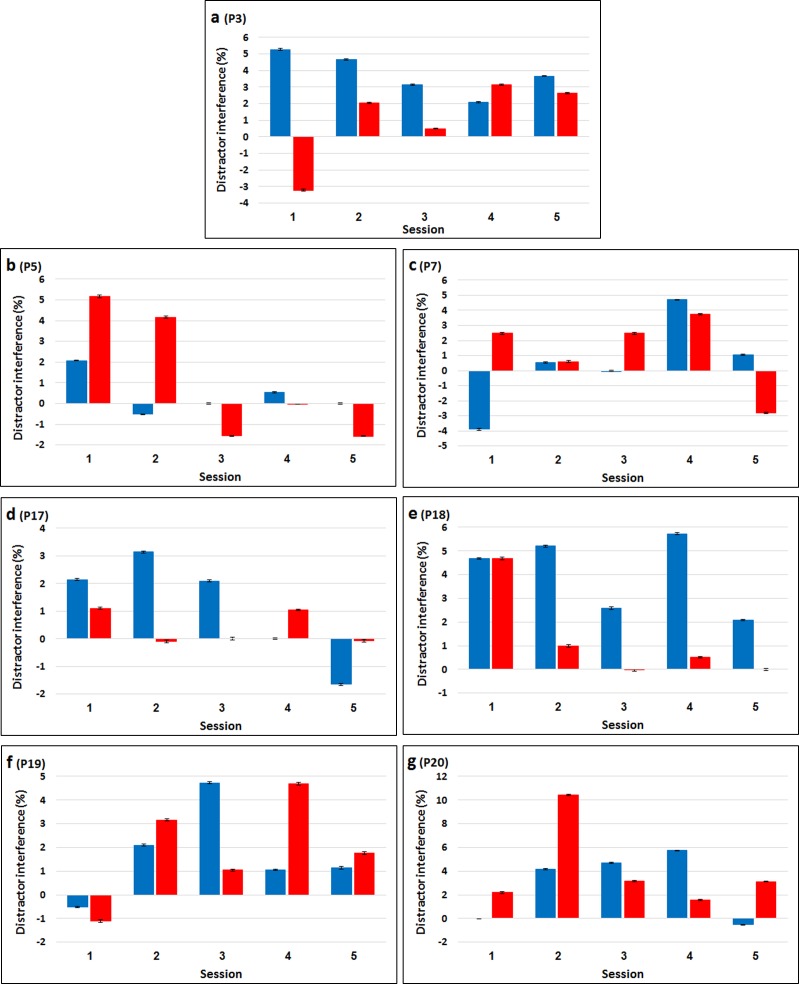
Accuracy distractor interference (incompatible vs. neutral) as a function of load condition and session for each of the seven participants. Error bars represent 95% confidence intervals (bootstrapped).

The RTs and accuracy of each one of these seven participants, in all five sessions, are presented in Table 4. Figs 10 and 11 present distractor interference (Incompatible RT—Neutral RT; Neutral accuracy—Incompatible accuracy) as a function of load condition for each of the sessions of each participant. As can be clearly seen, each of these seven participants performed very differently on the different sessions. No consistent pattern emerged. With all seven participants, the pattern of results followed the load theory on some of the sessions with one or both measurements. However, on other sessions the pattern was opposite to that predicted by the theory. In addition, each of the participants demonstrated in at least one session a reversed distractor effect. Although performance in terms of absolute values of RT and accuracy typically improved with session number (Table 4), likely reflecting general practice effects, no consistent pattern across sessions’ order emerged for distractor interference (Figs 10 and 11). That is, the participants do not seem to be more consistent in the last two sessions than in the first two sessions, nor do they tend to show patterns that are more/less similar to the theory's predictions, or to each other with later sessions. This lack of consistency suggests that the main factor mediating the individual differences found in all the different experiments described here is not some fixed personality trait or a well-established skill.

**Table 4:** Mean correct RT and accuracy as a function of session, load level, and compatibility condition, for each of the seven participants.

Participant	Session	RT (ms)				Accuracy (%)			
		Low load		High load		Low load		High load	
		Incomp.	Neutral	Incomp.	Neutral	Incomp.	Neutral	Incomp.	Neutral
p3	1	785	743	930	927	91.0	96.2	94.5	91.2
	2	685	684	773	764	92.7	97.3	93.7	95.8
	3	693	654	790	762	95.8	98.9	98.4	98.9
	4	647	617	764	720	95.8	97.9	95.3	98.4
	5	576	541	705	685	95.3	98.9	95.8	98.4
p5	1	643	641	786	790	96.9	98.9	87.0	92.1
	2	640	614	683	677	99.5	98.9	91.7	95.8
	3	586	576	660	649	98.9	98.9	99.5	97.9
	4	587	578	632	638	97.9	98.4	98.4	98.4
	5	524	492	574	567	98.4	98.4	99.5	97.9
p7	1	761	751	860	909	93.5	89.6	85.2	87.6
	2	866	776	902	937	91.9	92.4	88.1	88.7
	3	808	877	958	925	97.3	97.3	90.1	93.3
	4	780	726	862	839	92.1	96.8	91.0	94.8
	5	898	971	1025	969	95.5	96.5	95.0	92.2
p17	1	764	719	791	788	94.1	96.3	95.2	96.3
	2	758	733	863	829	93.7	96.8	93.7	93.6
	3	779	730	824	793	95.7	97.8	93.5	93.5
	4	598	575	635	644	99.0	99.0	98.4	99.5
	5	683	683	799	731	96.4	94.7	95.8	95.7
p18	1	659	635	806	832	94.3	99.0	89.3	94.0
	2	618	604	717	697	92.7	97.9	93.8	94.7
	3	583	569	660	659	92.7	95.3	94.8	94.8
	4	598	576	659	657	92.7	98.4	96.4	96.9
	5	588	574	626	622	95.3	97.4	96.9	96.9
p19	1	571	580	737	762	96.9	96.4	91.1	90.0
	2	598	564	716	693	94.8	96.9	91.1	94.2
	3	563	550	673	660	93.2	97.9	93.8	94.8
	4	520	510	643	641	97.4	98.4	90.1	94.8
	5	583	515	661	615	92.0	93.1	87.8	89.5
p20	1	527	498	732	748	95.8	95.8	77.0	79.2
	2	426	422	616	601	92.7	96.9	79.7	90.1
	3	470	473	611	601	91.1	95.8	92.7	95.8
	4	429	413	539	536	92.7	98.4	92.2	93.8
	5	433	437	554	540	97.4	96.9	90.6	93.8

## General discussion

This study was motivated by a recurrent difficulty to replicate the expected pattern of results obtained with the typical paradigms designed to assess distractor interference as a function of perceptual load. Although the methods of our experiments were quite similar to those employed by others who demonstrated the typical results, the expected pattern of results was observed in only some of our experiments, often only with one or the other performance measurement (i.e., RT or accuracy) but not both. Although most of the cases in which the findings were not in line with the load theory were observed with the accuracy measurement, this does not pose a smaller challenge for the theory as its predictions are not unique for RT, but rather refer to any performance measurement [e.g., 1, 2].

One possible explanation of this replication difficulty could be a large degree of individual differences that generates highly different results depending on the specific mixture of participants taking part in a given experiment. To test this possibility, we first reanalyzed the data of eight experiments that were already reported in the past [3, 4]. In these previous studies we only examined the aggregated performance. Here we focused on individual differences, examining separately for each participant the effect of load level on distractor interference. Looking at the data at the individual level revealed substantial inter-participants differences, with a particularly low percentage of participants whose performance matched the predictions of the load theory (i.e., larger distractor interference with low levels of perceptual load than with high levels of perceptual load). Specifically, with RT this percentage ranged between 5% and 44%, and with accuracy it ranged between 4% and 33%. Thus, even the highest percentage of participants whose performance fit the theory is still below 50%. This suggests that the large inter-individual variance found in these experiments cannot be easily dismissed as reflecting the noisy nature of our perceptual/cognitive system. That is, taking internal noise into consideration indeed predicts some variability in the data, however for a theory to be valid it has to predict that factors that are cardinal according to theory should have a larger effect on performance than noise, and therefore it has to predict that even when internal noise is considered, most of the participants should show an effect in the direction predicted by the theory. However, in all these experiments, and with both performance measurements, most of the observers did not show the expected pattern of results. It is hard to see how internal noise can help a theory deal with such a degree of inconsistency.

Importantly, this was the case even in the sole experiment with which the pattern of results at the aggregate level followed the predictions with both measurements (Experiment 4 of Marciano and Yeshurun [3]). Even with this experiment we found large deviation from the predicted pattern at the individual level (only 20% of the participants matched the theory with RT, and only 15% with accuracy). This suggests that the picture portrayed by the aggregated results obtained with this task does not tell the full story of the data. One explanation for this difference between the aggregated results and those observed at the individual level is that in all our experiments a reversed compatibility effect was often observed. That is, a low distractor interference at the aggregated level could indeed be found if most of the participants have only a small difference between the neutral and incompatible conditions. However, if many of the participants have a large difference between the incompatible and neutral conditions, only in the opposite direction to that expected, these participants will contribute negative values to the aggregated average, reducing its overall value as if the differences were all small. Hence, the finding that the aggregated analysis and that of the individual data do not lead to the same results, and particularly the frequent occurrence of a reversed compatibility effect, observed in these various experiments, stress the importance of examining individual-data in combination with aggregated results.

The findings of large inter-individual variance in all these experiments, and that the aggregated and individual analyses do not lead to the same results have very important implications regarding both the numerous previous studies that have used a version of this paradigm (see [21] for a recent review) as well as future studies that may use this paradigm, regardless of the theory they intend to examine, because these findings question the validity of any conclusion that is based on the aggregated data.

Considerable individual differences in the effect of perceptual load on distractor interference were reported previously by Fitousi and Wenger [14] (Experiment 1). They employed a similar paradigm to the one reported here, however their load manipulation was based on set-size variations. Fitousi and Wenger suggested that the individual differences they found might be due to differences in attention allocation. However, because 50% of their participants (four out of eight) exhibited a pattern of results that match the predictions of the load theory, and so did their aggregated data, they concluded that the cardinal hypotheses of the theory are supported. They further suggested that these inter-participants differences in attention allocation might be due to differences in processing capacity and therefore are in line with the theory. Indeed, as Fitousi and Wenger suggest, if a specific participant has particularly low perceptual capacity the theory predicts that he will demonstrate high level of attentional selectivity (i.e., no distractor interference), whereas a participant with relatively high perceptual capacity should demonstrate low selectivity or high interference with both load condition. Hence, the theory can accommodate a certain degree of individual differences. However, in all the experiments examined here, the percentage of participants whose pattern of results followed the theory was smaller than 50%, often considerably so. Moreover, frequently the pattern of results was opposite to that predicted by the theory–distractor interference was larger in the high load condition than in the low load condition. This pattern of results cannot be accounted for by the load theory even if different levels of perceptual capacity are assumed. Finally, the load theory cannot explain the often observed reversed compatibility effect (i.e., better performance in the incompatible than neutral condition, also found in the data of two participants of Fitousi & Wenger [14]), nor can any other theory we are aware of.

Like Fitousi and Wenger [14], other researchers have also embraced the hypothesis that inter-individual differences found with distractor-interference paradigms are due to differences in processing capacity (e.g., [18–22]). For instance, Green and Bavelier [18] attributed the fact that video-game players exhibit distractor processing under high levels of load to their high perceptual capacity. Similarly, Shipstead et al. [20] attributed differences in distractor interference under low levels of load to working memory capacity. Inter-individual differences in distractor interference as a function of perceptual load were also attributed to fixed personality traits, such as anxiety (e.g., [15, 16]) or absent-mindedness [23], or to enduring disorders such as autism [17].

This idea, that inter-individual differences are due to some stable trait or perceptual capacity motivated the second part of this study. In this part, 20 additional participants took part in a new experiment, which was identical to Experiment 4 in Marciano and Yeshurun [3] and very similar to Experiment 1 in Lavie and Cox [2]. Unlike these two previous experiments, with our new experiment a significant distractor interference under high level of load emerged with the accuracy measurement, and it was larger than the interference found with the low load condition. Hence, this new experiment constitutes another instance of a failure to replicate previously observed results regardless of methodological similarity. In contrast to this failure to replicate previous results at the aggregate level, we replicated the large individual differences observed in the eight experiments discussed in the first part of this study. Seven out of the 20 participants of the current Experiment took part in additional four identical experimental sessions. Three of them were chosen due to the fact that each one of them exhibited a different pattern of results in the first session. The other four participants were randomly assigned. We reasoned that if the observed individual differences are due to a fixed perceptual/cognitive capacity or a specific personality trait, then a relatively consistent pattern of results should emerge in all 5 experimental sessions of a given participant. However, in contrast to this prediction, high inconsistency between one session to the other was found with all seven participants, and this was the case with both performance measurements—RT and accuracy.

As discussed above, the perceptual load theory cannot account for the inter-individual differences we found in the first part of this study, at least not all the cases in which there was larger distractor interference with the high than low load condition or when there was a reversed compatibility effect. The high intra-individual differences found in the second part also pose a challenge to the theory. This is because the load levels were constant in all experimental sessions. Even if one is ready to assume that there were very large variations in perceptual capacity for a given participant between the different experimental sessions, still the load theory cannot account for the sessions in which we found an opposite effect of load or a reversed compatibility effect. Besides, if perceptual capacity of a given participant varies to such a large degree from one experimental session to the other, then the critical factor that mediates distractor interference is not perceptual capacity but rather the factor that determines capacity in each session.

It is important to note that large inter- and intra-individual variance is not common to all tasks that examine attentional effects, not even to tasks such as the Stroop and Flanker tasks that, like the current paradigm, look at distraction effects of irrelevant information. For instance, regarding inter-individual variance, several studies that examined individual differences in allocation of spatial attention based on spatial precues report some variance with various perceptual tasks, as expected given a noisy system, still most of the participants in these studies demonstrated the expected effects of attentional cueing (e.g., [24–27]). Likewise, Bender and colleagues [28] examined individual differences with several attention-related tasks including the attentional blink paradigm, the Stroop task and the Flanker task. They, too, find a certain degree of inter-individual differences, however with all of these tasks most of their participants showed the expected pattern of results. As for intra-individual variance, Huang, Mo and Li [29] examined many different tasks. They report high internal reliability for 16 out of their 21 attention-related tasks, and these 16 tasks include the Stroop task. Additionally, Bender et al. [28], reported high internal reliability for the Flanker task. Hence, high intra-participants variability is not a typical characteristic of attention related tasks, and seems to be more the exception than the rule.

In the recent years, alternative explanations to the load theory were offered to account for the pattern of results often observed with distractor-interference paradigms. For instance, Tsal and Benoni (e.g., [7, 8]) suggested that the typical lack of interference under high levels of load is due to the effect of dilution. The additional neutral letters that share features with the target and distractor dilute the interference effect brought about by the incompatible distractor. In support of this claim they found that when the additional neutral letters were different in colour from the target, ensuring that the search for the target is easy (i.e., low load), there was no distractor interference. Hence, even though in this condition the perceptual load was low, the mere addition of diluting items (i.e., neutral letters) eliminated the interference. Another alternative factor that may mediate distractor interference is distractor saliency (e.g., [6]). For example, Biggs and Gibson [30] examined the degree in which a salient distractor generated interference under varying levels of perceptual load. They found that under certain task context conditions, such as when load level was fixed for a given block, saliency was a more cardinal factor than perceptual load. The 'slippage theory' provides another alternative explanation [31]. According to this theory, distractor interference reflects the fact that on some of the trials observers mistakenly allocate spatial attention to the distractor, and such misplaced attention allocation is more likely under low levels of load because less target-like items are competing for attention allocation. The theory is supported by the findings that cueing the target, a manipulation expected to reduce the likelihood of mistakenly attending the distractor, considerably reduces distractor interference, while cueing the distractor considerably increased interference. Notwithstanding the relevance of these alternative explanations, they also cannot account for the inter-individual and intra-individual differences found here, because the display characteristics and task context were constant for all the participants of a given experiment and in all experimental sessions of a given participant. Thus, the factor mediating the large performance variations observed here cannot be something that is inherent to the stimuli or task, as suggested by these accounts.

Another alternative account of performance in this task suggests that the extent of the attentional focus (i.e., whether it is relatively wide or narrow) modulates the degree of distractor processing (e.g., [7, 32, 33]1). According to this account, regardless of perceptual load, a narrow focus of attention will reduce distractors processing in comparison to a wide spread of attention. Evidence that supports this account include, among others, the finding that when attention can be narrowly focused due to a precue that indicates the target location with 100% validity, the effect of load level is eliminated [34]. Another example is the finding that the processing of non-target items was limited when the participants knew in advance that the target could only appear in one of two locations in comparison to when it could appear in one of six locations [32]. Hence, the processing of irrelevant items was reduced when attention could be focused over a narrower region. The original framework of this account attributes the extent of the attentional focus to display characteristics or task settings. Within this framework this account also fails to explain the inter- and intra-individual differences described here. However, the current findings could be accommodated by this account if its original framework is extended to include changes in attentional focus that are not due to external factors but rather depend on internal states such as cognitive strategy. For example, the observed differences in distractor interference can be attributed to the extent of the attentional focus if one is ready to assume that: (a) different participants may adopt different strategies regarding the extent of their attentional focus, even when all external conditions (e.g., load level, prior knowledge, task settings) are identical; and (b) a given participant may adopt, on different experimental sessions, different strategies regarding the extent of her attentional focus, even when all external conditions in all sessions are identical. Still, this would leave open the question of which internal factors/states lead to a narrow focus of attention and which lead to a broader spread of attention.

Finally, in the past we considered a more active view of the attentional selectivity, with which distractor interference is prevented via an active inhibition of irrelevant stimuli [3,4]. We postulated that this inhibitory mechanism is only activated when processing demands are relatively high. That is, when task demands are relatively low (e.g., when perceptual load is low), there is no need to actively inhibit irrelevant items because the task can be accomplished to a satisfactory level even if the distractor is processed. But when task demands are moderately hard, adequate performance requires active inhibition of irrelevant items, because under such conditions perceiving the distractor might have a detrimental effect on performance. Still, if the task is particularly hard the participants may not have spare resources to invest in inhibition, and irrelevant items will be processed. This active view of the attentional selection can accommodate inter-individual differences because it seems reasonable to assume that different people may view similar task settings as posing different challenges. That is, under similar 'external' conditions one participant may find the task demands 'worth' the investment in active inhibition while another participant may manage to perform at an appropriate level without needing to activate the inhibitory mechanism. Hence, different patterns of distractor interference may be found for different participants.

However, as was the case with the attentional focus account, to accommodate the intra-individual differences found in the second part of the current study we will have to also assume that the activation of the inhibitory mechanism can depend solely on internal factors. Once again, the critical question that still remains is what are these internal factors? At this stage we can only speculate. One such internal factor that is already known to be involved in distractor processing is acute stress. Sato, Takenaka and Kawahara [35] found that participants who had to deliver a stressful oral presentation demonstrated an opposite pattern of distractor interference as a function of load (i.e., larger interference under high than low perceptual load). Although we cannot rule it out, we do not think that such acute stress is the factor mediating the large intra-individual differences we found in the second part of this study, and particularly for participants 17–20 of the current experiment. All experimental sessions were conducted within a relative short time interval in a period of the academic year that is not particularly stressful. We do not have access to the participants’ personal experiences and we therefore cannot provide evidence to support or rule out the possibility that their levels of stress varied considerably between experimental sessions. However, given that all seven participants, including the four participants with no selection criteria for whom we carefully controlled the time intervals between sessions and the time of the day at which the session was held, demonstrated considerable variations in the pattern of distractor processing between the different sessions, we find this possibility less likely.

Another candidate is fatigue. Csatho et al. [36] employed the typical distractor-interference paradigm and varied the levels of load by changing set-size. Critically, their participants perform the task for 2.5 hours without a break. They found that distractor interference increased with increasing levels of fatigue for the low load condition, but decreased with increasing levels of fatigue for the high load condition. Given this finding it is reasonable to assume that different individuals who come to the same experiment with different levels of fatigue will show different patterns of load effect on distractor interference. Similarly, it is possible that the same participant may come to different experimental sessions with different levels of fatigue, and therefore will show the large performance variance observed here. Because Csatho et al. [36] found that fatigue leads to increased distractor interference with low load and decreased interference with high load, their finding suggests that distractor interference should always be higher in the low than high load condition, in line with the load theory. Hence, their effects of fatigue cannot explain our findings of an opposite distractor interference or that of reversed compatibility effect. Nevertheless, we find fatigue as a likely factor mediating the large inter- and intra-individual differences found here, and we are currently exploring in our lab different types of fatigue.

To sum, we found large variations between different participants in their pattern of distractor interference as a function of load, and this was the case with 8 experiments reported in previously published papers and a new experiment reported here. We also found similar large variations in the pattern of results of a given participant in 5 identical experimental sessions. Both inter- and intra-individual variations included patterns of results that follow the predictions of the load theory, were opposite to these predictions, or revealed a reversed compatibility effect. None of the theories suggested thus far to account for performance with the typical distractor interference paradigm can explain these results. Particularly challenging are the large intra-individual variations and the reversed compatibility effect. The only conclusion one can draw, at this point, is that the paradigm described in the current study should be used very cautiously as we do not know which factors mediate performance and therefore our ability to reach any theoretical conclusion based on performance with this paradigm is very limited.

## Supporting information

S1 File Appendices A and B. (DOCX)
