# Supplementary material for: Large inter-individual and intra-individual variability in the effect of perceptual load
Source: PLoS One. 2017 Apr 13;12(4):e0175060. doi: 10.1371/journal.pone.0175060 (PMC5391011; doi:10.1371/journal.pone.0175060)
Supplement: S1 File — (DOCX) [file pone.0175060.s001.docx]

**Appendix A: Full description of the results of Marciano and Yeshurun [3]**

**Experiments 1, 2a and 2b**

A three-way repeated measures ANOVA, Central load (low vs. high), Peripheral load (none, low, and high), Compatibility (incompatible vs. compatible) was conducted on mean correct RT and accuracy data. RTs shorter than 100 ms or longer than 2000 ms were excluded from the analyses. All significant relevant main effects and interaction are presented in Table 5 and the mean correct RT and accuracy data are presented in Table 6. We did not include effects that are related to peripheral load because the corresponding data was not reanalyzed in the current study.

**Experiments 3 and 4**

A two-way repeated measures ANOVA, Central load (low vs. high) x Compatibility (incompatible, compatible, and neutral) was conducted on mean correct RT and accuracy data. RTs shorter than 100 ms or longer than 2000 ms were excluded from the analyses. All significant relevant main effects and interaction are presented in Table 5 and the mean correct RT and accuracy data are presented in Table 6.

**Table 5:** Significant effects of the ANOVAs (central load x compatibility) of all the experiments in Marciano and Yeshurun [3].

| **Exp.** | **Measurement** | **Effect** | **df** | **F** | **p** | **η_p_^2^** |
| --- | --- | --- | --- | --- | --- | --- |
| 1 | RT | central load | 1, 18 | 389.17 | p<0.0001 | 0.96 |
|  |  | central load x compatibility | 1, 18 | 5.18 | p<0.04 | 0.22 |
|  | Accuracy | central load | 1, 18 | 85.32 | p<0.0001 | 0.96 |
|  |  | compatibility | 1, 18 | 5.65 | p<0.03 | 0.11 |
| 2a | RT | central load | 1, 23 | 177.36 | p<0.0001 | 0.89 |
|  |  | compatibility | 1, 23 | 7.45 | p<0.02 | 0.33 |
|  | Accuracy | central load | 1, 23 | 70.33 | p<0.0001 | 0.75 |
|  |  | compatibility | 1, 23 | 3.43 | p=0.077 | 0.22 |
| 2b | RT | central load | 1, 19 | 270.48 | p<0.0001 | 0.93 |
|  |  | compatibility | 1, 19 | 9.69 | p<0.006 | 0.34 |
|  | Accuracy | central load | 1, 19 | 72.76 | p<0.0001 | 0.79 |
|  |  | compatibility | 1, 19 | 13.61 | p<0.002 | 0.42 |
| 3 | RT | central load | 1, 19 | 144.16 | p<0.0001 | 0.88 |
|  |  | compatibility | 2, 38 | 24.81 | p<0.0001 | 0.57 |
|  | Accuracy | central load | 1, 19 | 59.49 | p<0.0001 | 0.74 |
|  |  | compatibility | 2, 38 | 30.91 | p<0.0001 | 0.62 |
| 4 | RT | central load | 1, 19 | 74.49 | p<0.0001 | 0.80 |
|  |  | compatibility | 2, 38 | 7.73 | p<0.002 | 0.29 |
|  |  | central load x compatibility | 2, 38 | 7.55 | p<0.002 | 0.28 |
|  | Accuracy | central load | 1, 19 | 89.06 | p<0.0001 | 0.82 |
|  |  | compatibility | 2, 38 | 7.35 | p<0.003 | 0.28 |

**Table 6:** Mean correct RT, accuracy, and the magnitude of the distractor interference as function of load and compatibility conditions (incompatible vs. compatible, in Experiment 1 or incompatible vs. neutral, in all other experiments) for all the experiments of Marciano and Yeshurun [3].

| **Experiment** | **Measurement** | **Load** | **Incompatible** | **Neutral/Compatible** | **Distractor interference magnitude** |
| --- | --- | --- | --- | --- | --- |
| 1 | RT (ms) | Low | 521 | 498 | 23 |
|  |  | High | 756 | 766 | -10 |
|  | Accuracy (%) | Low | 96.5 | 96.7 | 0.2 |
|  |  | High | 86.5 | 90.3 | 3.8 |
| 2a | RT (ms) | Low | 501 | 483 | 18 |
|  |  | High | 719 | 712 | 7 |
|  | Accuracy (%) | Low | 96.5 | 96.2 | -0.3 |
|  |  | High | 84.7 | 86.5 | 1.8 |
| 2b | RT (ms) | Low | 514 | 496 | 18 |
|  |  | High | 702 | 694 | 8 |
|  | Accuracy (%) | Low | 94.2 | 97.0 | 2.8 |
|  |  | High | 86.1 | 89.3 | 3.2 |
| 3 | RT (ms) | Low | 558 | 542 | 16 |
|  |  | High | 718 | 697 | 21 |
|  | Accuracy (%) | Low | 93.8 | 95.8 | 2 |
|  |  | High | 88.1 | 91.8 | 3.7 |
| 4 | RT (ms) | Low | 578 | 559 | 19 |
|  |  | High | 737 | 735 | 2 |
|  | Accuracy (%) | Low | 95.2 | 97.6 | 2.4 |
|  |  | High | 89.3 | 90.4 | 1.1 |

**Appendix B: Full description of the results of Yeshurun and Marciano [4]**

A two-way repeated measures ANOVA, Central load (low vs. high) x Compatibility (incompatible, compatible, and neutral) was conducted on mean correct RT and accuracy data. RTs shorter than 100 ms or longer than 2000 ms were excluded from the analyses. All significant relevant main effects and interaction are presented in Table 7 and the mean correct RT and accuracy data are presented in Table 8.

**Table 7:** Significant effects of the ANOVAs (central load x compatibility) of all the experiments in Yeshurun and Marciano [4].

| **Exp.** | **Stimuli Duration** | **Measurement** | **Effect** | **df** | **F** | **p** | **η_p_^2^** |
| --- | --- | --- | --- | --- | --- | --- | --- |
| 2 | 100 | RT | central load | 1, 23 | 18.17 | p<0.0004 | 0.44 |
|  |  |  | central load x compatibility | 2, 46 | 5.91 | p<0.006 | 0.20 |
|  |  | Accuracy | central load | 1, 23 | 35.43 | p<0.0001 | 0.90 |
|  |  |  | compatibility | 2, 46 | 5.68 | p<0.007 | 0.64 |
|  |  |  | central load x compatibility | 2, 46 | 2.70 | p=0.0776 | 0.11 |
|  | 150 | RT | central load | 1, 23 | 52.71 | p<0.0001 | 0.70 |
|  |  |  | compatibility | 2, 46 | 4.90 | p<0.02 | 0.18 |
|  |  |  | central load x compatibility | 2, 46 | 4.18 | p<0.03 | 0.15 |
|  |  | Accuracy | central load | 1, 23 | 117.23 | p<0.0001 | 0.84 |
|  |  |  | compatibility | 2, 46 | 27.88 | p<0.0001 | 0.55 |
| 3 | 100 | RT | central load | 1, 17 | 100.94 | p<0.0001 | 0.86 |
|  |  |  | compatibility | 2, 34 | 29.59 | p<0.0001 | 0.64 |
|  |  |  | central load x compatibility | 2, 34 | 3.94 | p<0.03 | 0.19 |
|  |  | Accuracy | central load | 1, 17 | 36.72 | p<0.0001 | 0.68 |
|  |  |  | compatibility | 2, 34 | 12.21 | p<0.0001 | 0.42 |
|  | 150 | RT | central load | 1, 17 | 77.87 | p<0.0001 | 0.82 |
|  |  |  | compatibility | 2, 34 | 23.65 | p<0.0001 | 0.58 |
|  |  | Accuracy | central load | 1, 17 | 39.75 | p<0.0001 | 0.70 |
|  |  |  | compatibility | 2, 34 | 14.70 | p<0.0001 | 0.46 |
| 4 | 100 | RT | central load | 1, 17 | 11.88 | p<0.004 | 0.41 |
|  |  |  | compatibility | 2, 34 | 12.46 | p<0.0001 | 0.42 |
|  |  |  | central load x compatibility | 2, 34 | 3.41 | p<0.05 | 0.17 |
|  |  | Accuracy | central load | 1, 17 | 70.89 | p<0.0001 | 0.81 |
|  |  |  | compatibility | 2, 34 | 67.65 | p<0.0001 | 0.80 |
|  | 150 | RT | central load | 1, 17 | 70.89 | p<0.0001 | 0.34 |
|  |  |  | compatibility | 2, 34 | 67.65 | p<0.0001 | 0.21 |
|  |  | Accuracy | central load | 1, 17 | 74.53 | p<0.0001 | 0.81 |
|  |  |  | compatibility | 2, 34 | 26.52 | p<0.0001 | 0.61 |

**Table 8:** Mean correct RT, accuracy, and the magnitude of the distractor interference as function of load and compatibility conditions (incompatible vs. neutral) for all the experiments in Yeshurun and Marciano [4].

| **Experiment** | **Measurement** | **Stimuli Duration (ms)** | **Load** | **Incompatible** | **Neutral** | **Distractor interference**  **magnitude** |
| --- | --- | --- | --- | --- | --- | --- |
| 2 | RT (ms) | 100 | Low | 624 | 608 | 16 |
|  |  | 100 | High | 665 | 676 | -11 |
|  |  | 150 | Low | 611 | 600 | 11 |
|  |  | 150 | High | 701 | 699 | 2 |
|  | Accuracy (%) | 100 | Low | 83.4 | 89.1 | 5.7 |
|  |  | 100 | High | 65.1 | 74.8 | 9.7 |
|  |  | 150 | Low | 87.4 | 91.6 | 4.2 |
|  |  | 150 | High | 72.3 | 78.0 | 5.7 |
| 3 | RT (ms) | 100 | Low | 612 | 574 | 38 |
|  |  | 100 | High | 726 | 713 | 13 |
|  |  | 150 | Low | 607 | 581 | 26 |
|  |  | 150 | High | 735 | 709 | 26 |
|  | Accuracy (%) | 100 | Low | 93.3 | 95.9 | 2.6 |
|  |  | 100 | High | 86.9 | 89.9 | 3 |
|  |  | 150 | Low | 94.4 | 96.1 | 1.7 |
|  |  | 150 | High | 87.3 | 90.3 | 3 |
| 4 | RT (ms) | 100 | Low | 657 | 643 | 14 |
|  |  | 100 | High | 679 | 690 | -11 |
|  |  | 150 | Low | 665 | 642 | 23 |
|  |  | 150 | High | 693 | 689 | 4 |
|  | Accuracy (%) | 100 | Low | 80.3 | 88.7 | 8.4 |
|  |  | 100 | High | 64.4 | 75.1 | 10.7 |
|  |  | 150 | Low | 88.7 | 92.5 | 3.8 |
|  |  | 150 | High | 73.1 | 79.6 | 6.5 |
